# Impaired distensibility of ascending aorta in patients with HIV infection

**DOI:** 10.1186/1471-2334-12-167

**Published:** 2012-07-30

**Authors:** Alexandra Zormpala, Nikolaos V Sipsas, Ioannis Moyssakis, Sarah P Georgiadou, Maria N Gamaletsou, Athanasios N Kontos, Panayiotis D Ziakas, Theodore Kordossis

**Affiliations:** 1 Radiology Department, Laikon General Hospital of Athens, Medical School, National and Kapodistrian University, Mikras Asias 75, 11527, Athens, Greece; 2 Infectious Diseases Unit, Pathophysiology Department, Laikon General Hospital, Medical School, National and Kapodistrian University, Mikras Asias 75, 11527, Athens, Greece; 3 Cardiology Department, Laikon General Hospital of Athens, Mikras Asias 75, 11527, Athens, Greece

**Keywords:** HIV, Aortic distensibility, HAART, Atherosclerosis, Carotid artery intima-media thickness

## Abstract

**Background:**

Our aim was to investigate the aortic distensibility (AD) of the ascending aorta and carotid artery intima-media thickness (c-IMT) in HIV-infected patients compared to healthy controls.

**Methods:**

One hundred and five HIV-infected patients (86 males [82%], mean age 41 ± 0.92 years), and 124 age and sex matched HIV-1 uninfected controls (104 males [84%], mean age 39.2 ± 1.03 years) were evaluated by high-resolution ultrasonography to determine AD and c-IMT. For all patients and controls clinical and laboratory factors associated with atherosclerosis were recorded.

**Results:**

HIV- infected patients had reduced AD compared to controls: 2.2 ± 0.01 vs. 2.62 ± 0.01 10^-6^ cm^2^ dyn^-1^, respectively (p < 0.001). No difference was found in c-IMT between the two groups. In multiadjusted analysis, HIV infection was independently associated with decreased distensibility (beta –0.45, p < 0.001). Analysis among HIV-infected patients showed that patients exposed to HAART had decreased AD compared to HAART-naïve patients [mean (SD): 2.18(0.02) vs. 2.28(0.03) 10^-6^ cm^2^ dyn^-1^, p = 0.01]. In multiadjusted analysis, increasing age and exposure to HAART were independently associated with decreased AD.

**Conclusion:**

HIV infection is independently associated with decreased distensibility of the ascending aorta, a marker of subclinical atherosclerosis. Increasing age and duration of exposure to HAART are factors further contributing to decreased AD.

## Background

The use of highly active antiretroviral therapy (HAART) resulted in a significant decrease in morbidity and mortality in patients with human immunodeficiency virus (HIV) infection [1], turning this lethal infection to a chronic ambulatory disease. However, the initial optimism has been tempered when it became evident that HAART has metabolic side effects such as fat redistribution, dyslipidemia, insulin resistance, glucose intolerance, metabolic syndrome, and overt diabetes; all of them are established risk factors for atherosclerosis and cardiovascular disease [2-5]. Indeed, soon after the introduction of HAART into the clinical practice, researchers reported unexpected vascular events among young patients. A large prospective study confirmed that HAART, especially the protease inhibitor (PI)–containing regimens, increases the risk for cardio- and cerebro-vascular events in HIV-infected persons [6]. This finding suggests that HAART causes early atherosclerosis [7,8]. The observed excess cardiovascular risk cannot be attributed solely to the side effects of antiretroviral drugs since chronic HIV infection itself has a role, as it has been shown for other chronic inflammatory diseases [9]. Moreover, HAART increased life expectancy and as HIV seropositive population ages, chronic diseases like atherosclerotic cardiovascular disease become increasingly important [10,11].

It is difficult to dissect relative contributions of conventional cardiovascular risk factors, metabolic side effects of antiretroviral drugs, and HIV infection itself on early atherosclerosis and cardiovascular events, as these factors frequently co-exist in the same patient. Moreover, studies using clinical endpoints to investigate cardiovascular outcomes in patients with HIV infection, who typically are young or middle-aged, need large numbers of patients because of the low event rate. Therefore, studies using surrogate markers for early atherosclerosis are required.

Researchers have reported that the reduction of the elastic properties of aorta represents an early stage in the atherosclerotic process [12-16]. Aortic distensibility (AD) is an elasticity index of the ascending aorta, and along with carotid artery intima-media thickness (c-IMT) are simple and reproducible markers of subclinical arteriosclerotic disease and have been identified as strong predictors of cardiovascular mortality in different clinical settings [17-24]. There are no studies for early atherosclerosis of ascending aorta among HIV-infected patients. The aim of this study was to investigate the distensibility of the ascending aorta and c-IMT in HIV-infected patients compared with age and sex matched uninfected controls, and to investigate whether HIV infection itself, conventional risk factors for atherosclerosis, and/or HAART are associated with early atherosclerosis.

## Methods

We enrolled in the study a total of 105 consecutive, HIV-infected patients attending the outpatient clinic of the Athens Laikon Hospital. Controls were 124 healthy volunteers recruited from hospital staff, as well as their relatives or friends. HIV infection was ruled out in controls by serologic testing with their consent. Control subjects were individually matched with patients by age (± 5 years) and sex.

Demographic and clinical data such as age, sex, body weight, arterial blood pressure, and history of smoking were obtained from all patients and control subjects, as well as blood samples for laboratory measurements, including blood count, glucose, total cholesterol, triglyceride, high density lipoprotein (HDL), low-density-lipoprotein (LDL) and creatinine levels. Hypertriglyceridemia and hypercholesterolemia were defined as triglyceride and cholesterol level equal or more than 150 and 200 mg/dl, respectively. Hypertension was defined as systolic and diastolic blood pressure level above 140mmHg and 90mmHg, respectively. Moreover, for each HIV-infected patient the following information corresponding to the sampling time point was recorded: risk group, disease duration, CDC stage, CD4 cell count, viral load, and HAART. The study was approved by the Institutional Review Board of the Laikon General Hospital, Athens, Greece. All participants gave their informed consent.

All HIV-infected patients and seronegative controls were evaluated to determine c-IMT and AD. Visualization of the carotid artery was obtained via high resolution, B-mode carotid artery ultaronography, and c-IMT was measured by the same investigator. Measurement of c-IMT was performed in the common carotid artery, of both left and right side, 1cm proximal to carotid bulb and at least three separate measurements of each side were obtained according to previous recommendations [25]. Abnormal c-IMT was defined as a value of equal or more than 0.9 mm. AD was determined noninvasively based on the relationship between changes in aortic diameter and pressure with each cardiac pulse [26,27]. The echocardiographic study was carried out using a Hewlett Packard Sonos 1000 ultrasound system (Hewlett Packard), using a 2·5-MHz transducer. Each subject was placed in the mild left recumbent position and the ascending aorta was recorded at a level 3 cm above the aortic valve in the M-mode tracings guided by the two-dimensional echocardiogram in the parasternal long axis view [26]. Internal aortic diameters were measured by means of a caliper in systole and diastole as the distance between the trailing edge of the anterior aortic wall and the leading edge of the posterior aortic wall. Systolic aortic diameter was measured as the maximal anterior motion of the aorta and diastolic diameter at the peak of the QRS complex on the simultaneously recorded electrocardiogram. Ten consecutive cardiac beats were measured routinely and averaged [26,27]. Blood pressure was measured with a Dinamap TM XL vital signs monitor (Johnson-Johnson, Arlington, VA). AD was calculated according to the formula [26,28]:

(1) $$\text{Aortic distensibility}=\frac{2\Delta D}{Dd\;\left(Ps-Pd\right)}10^{-6}{\text{dyn}}^{-1}{\text{cm}}^{2}$$

Where *ΔD* is the change of the aortic diameter between systole and diastole, *Dd* is the aortic diameter in diastole, *Ps* is the systolic arterial blood pressure and *Pd* is the diastolic arterial blood pressure. The cardiologist who performed the measurements (IM) was blind of the results of the autonomic function tests of the examined subjects. The intraobserver and interobserver mean percentage error (absolute difference between two observations divided by the mean and expressed as percentage) was determined for the aortic dimensions in 20 randomly selected subjects and were 4.2% and 4.6% for the systolic and 4.1% and 4.4% for the diastolic dimensions in our centre, respectively.

## Statistical analysis

STATA package v8 (Stata Corporation, College Station, TX, USA) was used for data analysis. Continuous variables are presented as mean ± SD, and compared using the *t*-test. Dichotomous variables are presented as frequency (%) and compared using the chi-square test. For HIV-infected patients vs. controls comparison, a multiadjusted analysis for AD (dependent variable) which was the major outcome was performed using the linear regression technique and all significant variables of the univariate analysis entered the model as independent covariates.

In a second step, a univariate and multivariate linear regression (using a stepwise, backward selection technique) were performed in HIV-positive group only to identify influential factors for AD. A two-sided P-value <0.05 was considered as statistically significant.

## Results

### Characteristics of the study population

The study population consisted of 105 HIV seropositive patients (86 male [82%]) with mean age ± SD, 41 ± 0.92 years and 124 control subjects (104 male [84%] with mean age 39.2 ± 1.03 years. Sixty out of the 105 HIV-infected patients (57%) had acquired immunodeficiency syndrome (AIDS) and 89 of them (85%) were receiving HAART. Patients’ clinical characteristics are shown on Table 1. The prevalence of dislipidemia was higher among HIV-infected patients, as they had higher fasting plasma concentrations of total cholesterol (p < 0.001), triglycerides (p < 0.001) and LDL (p = 0.08) and lower concentrations of HDL (p < 0.001), compared to HIV- seronegative controls. On the contrary, the prevalence of obesity [Body mass index (BMI) > 30 kg/m^2^] was significantly higher in control subjects than in HIV-infected patients (p < 0.001). The mean arterial pressure was higher among controls regarding both systolic and diastolic arterial pressure (p < 0.001); hypertension was statistically more frequent in controls than in HIV-infected patients [13, (10.5%) vs. 1, (1%), respectively, p = 0.003] (Table 2).

**Table 1:** Clinical data in HIV-infected patients

**Patients characteristics**	**N=105**
AIDS, (%)	60 (57)
HAART, (%)	89 (85)
Disease duration (median/range, months)	81/1-246
CD4 (cells/μL)	519 ± 346
Viral load (copies/ml)	
Median	80
Range	40 - 860000
Undetectable (%)	50 (48)
IV Drug abuse	3 (3)
Treatment with PIs, (%)	71 (68)
Treatment with NNRTIs, (%)	42 (40)
Treatment with NRTIs, (%)	87 (83)
Duration of treatment, (median/range, months)	
HAART	75/1-147
PIs	47/3-104
NNRTIs	16/1-64
NRTIs	66/1-173

AIDS, acquired immunodeficiency syndrome; HAART, highly active antiretroviral treatment; PI, protease inhibitor; NNRTI, non-nucleoside reverse transcriptase inhibitor; NRTI, nucleoside reverse transcriptase inhibitor; IV, intravenous.

Data are given as mean ± standard deviation.

**Table 2:** Comparison of the two groups (Univariable analysis)

	**Patients**	**Controls**	**P value**
	**(N=105)**	**(N=124)**	
*Basic Demographics*			
Male sex, (%)	86 (82)	104 (84)	0.69
Age (years)	41.0 ± 0.92	39.2 ± 1.03	0.19
Greek origin, (%)	99 (94)	110 (89)	0.13
*Metabolic profile*			
Obesity, (BMI > 30 kg/m^2^)	5 (4.8)	25 (20.2)	0.001
Diabetes, (%)	4 (3.8)	3 (2.5)	0.54
Glucose (mg dL^−1^)	93.5 ± 1.67	95.2 ± 2.13	0.55
Hypertriglyceridemia, (%)	64 (61)	36 (30)	<0.001
Triglycerides (mg dL^−1^)	234.3 ± 19.5	118.5 ± 6.1	<0.001
Hypercholesterolemia, (%)	98 (93)	51 (41)	<0.001
Total cholesterol (mg dL^−1^)	220.5 ± 5.84	195.8 ± 4.14	<0.001
LDL (mg dL^−1^)	135.1 ± 5.07	124.2 ± 3.59	0.08
HDL (mg dL^−1^)	41.9 ± 0.95	47.9 ± 1.20	<0.001
Smokers, (%)	47 (37.9)	59 (56.2)	0.36
*History/Laboratory values*			
Coronary Artery Disease/Stroke, (%)	1 (1)	6 (4.8)	0.09
Arterial Hypertension, (%)	1 (1)	13 (10.5)	0.003
-systolic (mmHg)	117.7 ± 0.91	123.5 ± 1.03	<0.001
-diastolic (mmHg)	72.8 ± 0.63	77.9 ± 0.78	<0.001
Hb values (mg dL^−1^)	13.4 ± 0.17	14.2 ± 0.15	<0.001
Creatinine (mg dL^−1^)	0.95 ± 0.01	1.08 ± 0.02	<0.001
CRP (mg dL^−1^)	5.0 ± 0.78	9.4 ± 2.25	0.09
*Cardiac Indexes*			
IMT right (mm)	0.59 ± 0.01	0.66 ± 0.08	0.41
IMT right ≥ 0.9mm	4(4)	9(7)	0.26
IMT left (mm)	0.62 ± 0.01	0.67 ± 0.08	0.59
IMT left ≥ 0.9mm	11(10)	9(7)	0.39
Distensibility (10^-6^ dyn^-1^ cm^2^)	2.20 ± 0.01	2.62 ± 0.01	<0.001

Data are given as mean ± standard deviation.

BMI, body mass index; LDL, low density lipoprotein; HDL, high density lipoprotein; Hb, hemoglobin; CRP, C-reactive protein; IMT, Intima-media thickness of carotid artery.

### Distensibility of the ascending aorta and HIV infection in the whole study population

We first compared HIV-infected patients with controls using *t*-test. AD was statistically lower in patients’ population than in uninfected controls: 2.2 ± 0.01 vs. 2.62 ± 0.01 10^-6^ cm^2^ dyn^-1^, respectively (p < 0.001) (Table 2). Nevertheless, no difference was found in c-IMT between the two groups.

In multiadjusted analysis, after adjustment for all significant confounders, HIV infection was independently associated with decreased distensibility (beta –0.45, p < 0.001) (Table 3). Other factors associated with decreased AD were, as expected, obesity and increasing diastolic pressure.

**Table 3:** Multivariate* linear regression analysis: the potential effect of various confounding factors on aortic distensibility in the total study population (HIV-infected persons and controls)

	**Beta coefficient**	**P-value**
Obesity (yes vs. no)	-0.053	0.05
Triglycerides (mg/dl)	-0.00006	0.36
Total Cholesterol (mg/dl)	-0.0003	0.09
Systolic pressure (mm Hg)	-0.002	0.08
Diastolic pressure (mm Hg)	-0.003	0.03
Hb values (gr/dl)	+0.008	0.13
Creatinine (mg/dl)	+0.07	0.19
HIV seropositivity (yes vs. no)	-0.45	<0.001

HIV, human immunodeficiency virus; Hb, hemoglobulin.

Beta coefficient expresses the mean difference in distensibility. Positive sign corresponds to protective effect while minus sign to decreased distensibility.

*All the significant variables of Table 2 (with p<0.05) were included. When the same characteristic was reported as both continuous and categorical, the continuous variable was preferred, (e.g. cholesterol in mg/dl vs. hypercholesterolemia).

### Distensibility of the ascending aorta in HIV-infected patients

**Figure 1:**
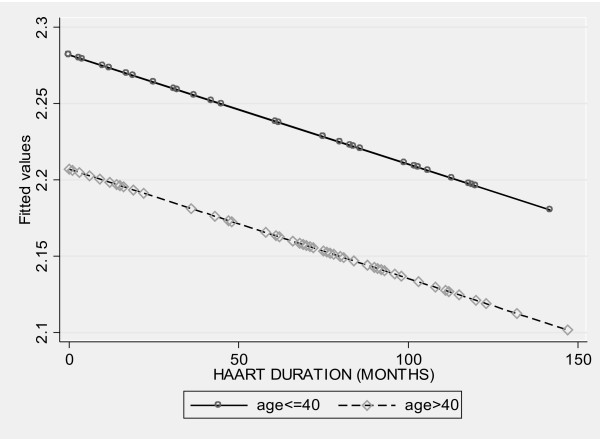
Effect of HAART therapy on the distensibility of ascending aorta, stratified for age < =40 years vs. >40 years old (fitted values).

A separate analysis was performed within the group of HIV-infected patients. Patients exposed to HAART had decreased distensibility of the ascending aorta compared to HAART-naïve patients [mean (SD): 2.18(0.02) vs. 2.28(0.03), p = 0.01]. In multivariate linear regression analysis only increasing age and cumulative duration of exposure to HAART were independently associated with decreased AD (Table 4). Based on the previous model, Figure 1 shows the effect of HAART therapy on the distensibility of the ascending aorta, stratified for age > =40 years vs. <40 years.

**Table 4:** Univariate & Multivariate* linear regression analysis: the potential effect of various confounding factors on aortic distensibility in HIV-infected patients

	**Univariate Analyis**		**Multivariate Analysis**	
	**Beta coefficient**	**p**	**Beta coefficient**	**p**
Age (per year)	-0.007	<0.001	-0.006*	<0.001
Female sex	+0.04	0.20		
Total Cholesterol	-0.0002	0.27		
Hemoglobin	+0.01	0.44		
CD4 (+) T-cell count	+0.00007	0.07		
Viral load (per 1000 copies increase)	+0.00004	0.76		
AIDS	-0.04	0.15		
HAART duration (months)	-0.002	0.004	-0.001*	0.04
HAART (exposed vs. naïve)	-0.1	0.01		
HIV duration (months)	-0.0005	0.02		

HIV, human immunodeficiency virus; AIDS, acquired immunodeficiency syndrome; HAART, highly active anti-retroviral therapy.

Beta coefficient expresses the mean difference in distensibility. Positive sign corresponds to protective effect while minus sign to decreased distensibility.

* after a stepwise, backward selection, using a p >=0.10 for variable exclusion.

## Discussion

The main findings of our study were that HIV-infected patients had significantly reduced distensibility of the ascending aorta compared to age and sex matched control subjects. On the contrary, no difference in c-IMT was observed between the two groups. Multiadjusted analysis showed that after adjustment for conventional cardiovascular risk factors HIV infection was independently associated with decreased AD. Among HIV-infected patients, those exposed to HAART had significantly decreased AD compared to HAART-naïve patients. In multivariate analysis, increasing age and duration of HAART exposure were factors independently associated with decreased AD. Moreover, the effect of HAART on the distensibility was more pronounced among patients older than 40 years old (Figure 1).

The reduced distensibility of the ascending aorta that we found among HIV-infected patients compared to uninfected controls is a marker of subclinical arteriosclerotic disease and has been identified as a strong predictor of cardiovascular mortality in different clinical settings [17-21]. In a large population study, AD was inversely related to conventional cardiovascular disease risk factors, such as older age, hypertension, smoking, and low HDL-cholesterol levels [29]. In accordance, in our study multiadjusted analysis showed that vascular risk factors such as obesity, and increasing diastolic pressure were also important for decreased distensibility of the ascending aorta. It is interesting that we found decreased distensibility among HIV-infected patients despite the fact that obesity and blood pressure were significantly higher among controls. One explanation could be that dislipidemia was statistically more frequent among HIV-infected patients, due to the HAART side effects or to the chronic infection *per se*.

Multi-adjusted analysis showed that after correction for other vascular risk factors, HIV infection was independently associated with decreased AD. In fact, premature atherosclerosis has been reported in young adults with HIV infection in the pre–HAART era [30]. The cardiovascular disease risk associated with HIV infection appears to be partially attenuated by antiretroviral treatment, since treatment interruption increases short-term risk of cardiovascular disease events [11]. These findings suggest that HIV infection itself may increase the risk for cardiovascular disease, as it has been shown for other chronic inflammatory diseases [9] in the non-HIV setting. Many markers of inflammation are markedly elevated in individuals with untreated HIV infection and are only partially reversed by effective combination antiretroviral therapy [31].

Our data are consistent with previous studies showing that HIV infection *per se*, as well as HAART is associated with increased stiffness [30,32-36] of peripheral arteries (femoral and branchial) and the carotid artery. Interestingly, recent investigations have shown that biological and vascular age in HIV infected patients is increased [37,38]. Our study did not find increased c-IMT in HIV-infected patients compared to uninfected controls. Conflicting evidence exists in the bibliography on the c-IMT: some studies reported increased c-IMT in HIV- infected patients compared with HIV-negative controls [30,33,39-44] but others did not [45-48]. It is noteworthy that our HIV-infected population had decreased distensibility of the ascending aorta and a normal c-IMT. This may imply that there are differences in the effect of HIV along the arterial system and reduced AD is an earlier marker of subclinical atherosclerosis compared to c-IMT. An alternative explanation could be that although arterial stiffening is thought to be a generalized phenomenon, different arterial segments are known to respond differently to atherosclerotic risk factors [49-51].

Our study showed that among HIV-infected patients, increasing age and longer exposure to HAART contribute further to decreased distensibility of the ascending aorta. Moreover, the effect of HAART on the AD was more pronounced among patients older than 40 years old. While increasing age is a well known risk for atherosclerosis, the effect of HAART on accelerating atherosclerosis remains controversial. Chronic HIV infection may lead to vascular endothelial damage and damage the elastic properties of an artery by sustaining a low degree of inflammation [52]. HAART has beneficial effects by reducing the inflammation due to active HIV infection, and detrimental effects such as dyslipidemia. Although it is difficult to determine the net effect in an individual patient and in different arterial segments, a recent study showed that endothelial dysfunction actually improved after start of HAART despite the rapid onset of dyslipidemia [53]. Our study had not enough statistical power to detect if PI containing regimens had different effect on the AD compared to NNRTI-containing regimens.

Our study has some limitations. Aortic diameters were measured echocardiographically and not invasively. A previous study has shown that aortic diameter can be determined with a high degree of accuracy in subjects whose cardiothoracic anatomy permits an echocardiographic signal of satisfactory quality, and the values obtained by echocardiography were not significantly different from those obtained by angiography [26]. In addition, pulse pressure estimated non-invasively from the brachial artery by external sphygmomanometry has been used for the calculation of AD in previous reports [26,54-56]. These non-invasive techniques have also been used for calculating AD in previous studies [55-57]. Thus a reliable estimation of the elastic properties of the ascending aorta using completely non-invasive techniques is feasible. Another limitation was the very high proportion of male subjects included in the cohort, which could limit the generalization of results to female HIV-infected patients. Moreover, the potential impact of past/active drug abuse could not be reliably evaluated, as only 3 patients (3%) had a history of drug abuse.

## Conclusions

HIV infection is independently associated with decreased distensibility of the ascending aorta, a marker of premature atherosclerosis. This suggests that patients with HIV infection may be at increased cardiovascular disease risk, independent of the presence of classical cardiovascular risk factors. Study of arterial elasticity as early marker of vascular damage could be promising and more appropriate investigation in HIV people than evaluation of cIMT.

## Competing interests

The authors declare that they have no competing interests.

## Authors’ contributions

AZ and NVS designed the study and drafted the study protocol with input from TK. SPG and MNG collected the data with input from ANK. IM performed the ultrasound measurements and contributed in the interpretation of data. PDZ conducted the data analysis. AZ and NVS wrote the manuscript. TK revised the final draft. All authors read and approved the final manuscript.

## Pre-publication history

The pre-publication history for this paper can be accessed here:

http://www.biomedcentral.com/1471-2334/12/167/prepub
